# β-Hydroxy-β-Methylbutyrate (HMB) Promotes Neurite Outgrowth in Neuro2a Cells

**DOI:** 10.1371/journal.pone.0135614

**Published:** 2015-08-12

**Authors:** Rafael Salto, Jose D. Vílchez, María D. Girón, Elena Cabrera, Nefertiti Campos, Manuel Manzano, Ricardo Rueda, Jose M. López-Pedrosa

**Affiliations:** 1 Department of Biochemistry and Molecular Biology II, School of Pharmacy, University of Granada, Granada, Spain; 2 Abbott Nutrition R&D, Granada, Spain; Université catholique de Louvain, BELGIUM

## Abstract

β-Hydroxy-β-methylbutyrate (HMB) has been shown to enhance cell survival, differentiation and protein turnover in muscle, mainly activating phosphoinositide-3-kinase/protein kinase B (PI3K/Akt) and mitogen-activated protein kinases/ extracellular-signal-regulated kinases (MAPK/ERK) signaling pathways. Since these two pathways are related to neuronal survival and differentiation, in this study, we have investigated the neurotrophic effects of HMB in mouse neuroblastoma Neuro2a cells. In Neuro2a cells, HMB promotes differentiation to neurites independent from any effects on proliferation. These effects are mediated by activation of both the PI3K/Akt and the extracellular-signal-regulated kinases (ERK1/2) signaling as demonstrated by the use of specific inhibitors of these two pathways. As myocyte-enhancer factor 2 (MEF2) family of transcription factors are involved in neuronal survival and plasticity, the transcriptional activity and protein levels of MEF2 were also evaluated. HMB promoted MEF2-dependent transcriptional activity mediated by the activation of Akt and ERK1/2 pathways. Furthermore, HMB increases the expression of brain glucose transporters 1 (GLUT1) and 3 (GLUT3), and mTOR phosphorylation, which translates in a higher protein synthesis in Neuro2a cells. Furthermore, Torin1 and rapamycin effects on MEF2 transcriptional activity and HMB-dependent neurite outgrowth support that HMB acts through mTORC2. Together, these findings provide clear evidence to support an important role of HMB in neurite outgrowth.

## Introduction

Neurite outgrowth is a requisite for an accurate functional network of neurons during development [1]. It is also crucial for neuronal plasticity [2], as well as neuronal regeneration [3]. The molecular mechanisms underlying the regulation of neurite outgrowth are well known. Activation of the extracellular-signal-regulated kinases (ERK1/2) [4, 5] and the phosphoinositide-3-kinase/protein kinase B (PI3K/PKB) [1, 6, 7] signaling pathways has been reported to regulate not only neuronal differentiation and survival but also several aspects of neurite outgrowth, including elongation, calibre and branching, Consequently, the finding of molecules that promote neurite outgrowth may improve brain development and maintain its function.

Among the transcription factors regulated by these signaling pathways that are involved in neuronal differentiation and survival stands out the Myocyte-enhancer factor 2 family (MEF2). This family of transcriptional regulators was first described in muscle development [8]. MEF2 isoforms A-D play key regulating roles in several cellular processes, including neuronal survival, differentiation, morphogenesis and apoptosis [8, 9] due to their over-expression in the brain during development [10–12]. The regulation of MEF2 activity is complex and it is mainly based in phosphorylation and dephosphorylation processes which in turn modulate their DNA-binding affinity, association with co-regulators, acetylation and sumoylation [12]. PI3k/Akt pathway is involved in the regulation of MEF2 activity upon either insulin-like growth factor-1 (IGF-I) stimulation or membrane depolarization [13].

The manipulation of both ERK and PKB/Akt activities with small, brain penetrant, neurotrophic molecules have potential to be beneficial for neuronal outgrowth, differentiation and plasticity. β-Hydroxy-β-methylbutyrate (HMB) is a leucine metabolite that has been used by its positive effects on muscle protein turnover, increasing protein synthesis and preventing protein degradation, in *in vitro* and *in vivo* models of muscle wasting [14–17]. HMB effects are mediated via the Mitogen-Activated Protein Kinases/ERK (MAPK/ERK) and PI3K/Akt pathways [18] [17]. However, the effect of HMB on neurons and its mechanism have not been yet described.

Therefore, the purpose of this study has been to assess the neurotrophic effects of HMB in mouse neuroblastoma Neuro2a cells. To the best of our knowledge, this is the first manuscript to report that HMB induces neurite outgrowth by mechanisms involving an increase in MEF2 levels and an activation of PI3K/Akt and ERK1/2 signaling pathways.

## Materials and Methods

### Materials

HMB free acid, rapamycin, LY294002 and PD98059 were from Sigma (St. Louis, MO, USA). Torin1 was from Selleck Chemicals (Houston, TX, USA). Tissue culture media, Fetal Bovine Serum (FBS) and supplements were from Sigma. ERK1/2 and phospho-ERK1/2 E10 (Thr202/Tyr204), PKB/Akt and phospho-PKB/Akt (Ser473), *mechanistic target of rapamycin* (mTOR) and phospho-mTOR (Ser2448) antibodies were from Cell Signaling Technology (Beverly, MA, USA). Glucose transporters 1 (GLUT1) and 3 (GLUT3), MEF2 and MEF2C antibodies were from Santa Cruz Biotechnology (Santa Cruz, CA, USA). MEF2D antibody was from BD Transduction Laboratories (San Diego, CA). A monoclonal antibody against Actin (JL20) was from Developmental Studies Hybridoma Bank (Iowa, USA). HRP-conjugated secondary antibodies were from BIO-RAD (Madrid, Spain).

### Cell culture

The murine neuroblastoma Neuro-2a (N2a; ATCC No. CCL-131) cell line was grown in Dulbecco’s Modified Eagle’s medium (DMEM) supplemented with 10% (v/v) FBS, 2mM glutamine plus 100 U/ml penicillin and 0.1 mg/ml streptomycin in an atmosphere of 5% CO_2_ and 95% air, and was maintained at sub-confluent densities in the growth media.

For cell proliferation experiments, DMEM supplemented with 10% FBS was used. For differentiation experiments, medium was replaced with DMEM supplemented with 1% FBS.

For the experiments using inhibitors for PKB/Akt (20 μM LY294002), ERK1/2 (10 μM PD98059) or mTOR (rapamycin 20 nM or Torin1 10 nM) mediated signaling, Neuro2a cells were treated with the appropriate inhibitor 30 min prior to 25 μM HMB administration and the inhibitor was maintained during the incubation time.

### Determination of protein synthesis

Protein synthesis was assesed by L-[ring-3,5-^3^H]-Tyrosine (Perkin Elmer, Waltham MA) as previously described by [19]. L-[ring-3,5-^3^H]-Tyrosine was added for 1 h to Neuro 2a cells that have preincubated for 2h in the absence (Control cells) or presence of 25 μM HMB (HMB cells) in DMEM with 10% FBS and 0.8 mM L-Tyrosine. Radioactivity in dissolved precipitates was counted using a scintillation counter (Beckman Coulter). Data was computed as dpm/μg of proteins.

### Cell counting and neurite outgrowth assays

Water soluble 3-(4,5-dimethylthiazol-2-yl)-2,5-diphenyl-2H-tetrazolium bromide (MTT) was added to the cells and bioreduced by dehydrogenases, which correlates the cellular metabolic activity with the number of viable cells in culture.

Neurite outgrowth was observed under a phase-contrast light microscope (Olympus CKX41) at a magnification of 200x. Images have been taken and the number of undiferentiated cells and neurites was counted using the program ImageJ (National Institutes of Health, USA). Neurites were defined as a process with lengths equivalent to one or more diameters of a cell body. The percentage of cells bearing neurites was calculated as the precentage of number of neurites divided by total number of cells.

### Acetylcholinesterase (AChE) activity assay and amount

AChE activity was determined by colorimetrically measuring the rate of hydrolysis of acetylthiocholine [20]. AChE antiboy was provided by Santa Cruz, AChE amount was determined by western blot as decribed below.

### Protein phosphorylation analysis

To study the phosphorylation status of the proteins involved in signaling events, Neuro2a cells were incubated in the presence or absence of 25 μM HMB for 30 min. Plates were flash frozen in liquid nitrogen and processed as described previously [21, 22].

To study the expression of GLUT1 and GLUT3 and MEF2 proteins, Neuro2a cells were incubated for 24 h in the presence or absence of 25 μM HMB. After treatment, they were lysed with RIPA buffer supplemented with phosphatase and protease inhibitors, 10 mM sodium fluoride, 10 mM sodium pyrophosphate, 1 mM sodium orthovanadate, 1 mM EGTA, 20 nM okadaic acid, 10 μg/ml aprotinin, 10 μg/ml leupeptin and 10 μg/ml pepstatin.

The protein concentration was measured using the bicinchoninic acid method [23]. Proteins (40 μg) were separated by sodium dodecyl sulfate polyacrylamide (SDS-PAGE), transferred onto nitrocellulose membranes, and immunoblotted with specific antibodies. To assay the phosphorylation degree of key kinases, antibodies against phosphorylated protein were used. To determine total levels of these kinases, independent western blots were carried out to avoid stripping of the proved membranes. Immunoblots were developed by an enhanced chemiluminescence detection method using a Chemidoc-it 810 Imager (UVP, Cambridge, UK). Quantification was performed by densitometry with the NIH Image Software [24].

### Reporter gene constructs and assays

For the analysis of the MEF2 dependent luciferase activity, cells at 80–90% confluence were transiently transfected with plasmid pMEF2X4-E1b-Luc (a kindly gift of Prof. Brian Black, UCSF, USA) using LipofectAMINE2000 as described by the manufactured. The DNA mixture comprised the luciferase reporter and the reference plasmid pRL-TK (ratio 95:5).

Neuro2a cells were incubated in presence or absence of 25 μM HMB for 24 h. Luciferase activity was determined using the Dual Luciferase method (Promega) in a luminometer (Sirius L, Berthold Technologies, Bad Wildbad, Germany) and results were standardized for Renilla luciferase activity. To allow comparison of the expression patterns, the data are expressed as relative changes in luciferase activity and were normalized to a value of 100%.

### Statistics

Results are expressed as mean ± standard error of the mean (SEM). Differences between mean values of control and HMB groups were analyzed by an unpaired t-test and between mean values of multiple groups by one way ANOVA followed by Tukey test using a bootstrap method for a small number of samples. P < 0.05 was considered statistically significant.

## Results and Discussion

In the present study, Neuro2a cells have been used as a well established model to evaluate the neurotrophic effects of HMB. It is a mouse neural crest-derived cell line that has been extensively used to study neural differentiation, neurite outgrowth [7, 25, 26], and cytotoxicity [27] and therefor, these neural cells have proven to be a useful model to study the effect of different molecules on neuron proliferation and differentiation.

**Fig 1:**
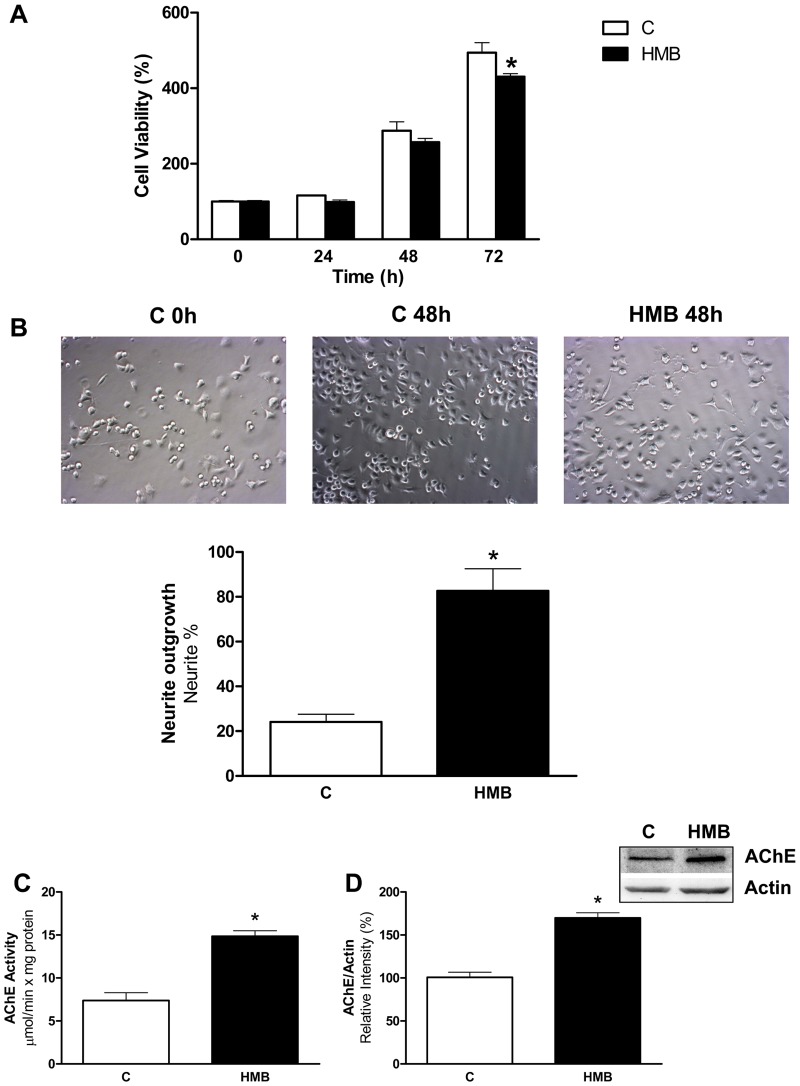
HMB induces neurite outgrowth in Neuro2a cells. **(A)** Neuro2A cells were incubated with 25 μM HMB during 72 h and cell viability was measured at different periods of time. **(B)** Neurite outgrowth was analyzed after 48 h incubation with HMB. Cell morphology was observed under a light microscope (200x) and the neurite bearing cells were counted. Results are plotted as percentage of neurite bearing cells. **(C)** Acetylcholinesterase activity was measured after 48 h incubation with HMB. **(D)** Acetylcholinesterase amount was measured by western blot using specific antibodies against AChE. after 48 h incubation with HMB. Results were normalized using actin as loading control. Results are expressed as means ± SEM (n = 4). * p<0.05 versus control cells.

First, we studied the effects of HMB on Neuro2a growth rate (Fig 1A) compared to control cells. 25 μM HMB was used as a the standard concentration used to study the effects of this molecule in muscle protein metabolism [17, 19]. HMB treatment had no effect on cell proliferation at 24 or 48 hours, although long-term treatment with HMB did have a small but significant effect on cell proliferation. This result is important since Neuro-2a cells have been described as a fast-growing mouse neuroblastoma cell line, and our results indicate that HMB has no unwanted proliferative effects on the cells in culture.

Next, we investigated whether HMB induces neurite outgrowth and differentiation. We analyzed morphological changes after 48 h of HMB treatment. As shown in Fig 1B, most of the untreated control cells showed round shape without neurite extension, whereas the formation of extensive neurites was observed in HMB-treated cells under phase contrast microscopy.

To further confirm HMB-induced neurite outgrowth in Neuro2a cells, AChE activity was measured as a marker of neurite formation [28, 29]. As shown in Fig 1C, HMB treatment of Neuro2a cells for 2 days increased two fold the AChE activity compared with untreated control cells. The changes in AChE activity are parallel to the changes in the amount of enzyme measured by western blot (Fig 1D). These results suggest that HMB effect on Neuro2a is based mainly on promoting neurite elongation and branching rather than increasing cell proliferation. An increase in neurite differentiation in the Neuro2a cell line has been proposed as a marker for improvement of the long-term memory formation [30], and therefore justify the elucidation of the molecular bases of the HMB on the neurite outgrowth process.

**Fig 2:**
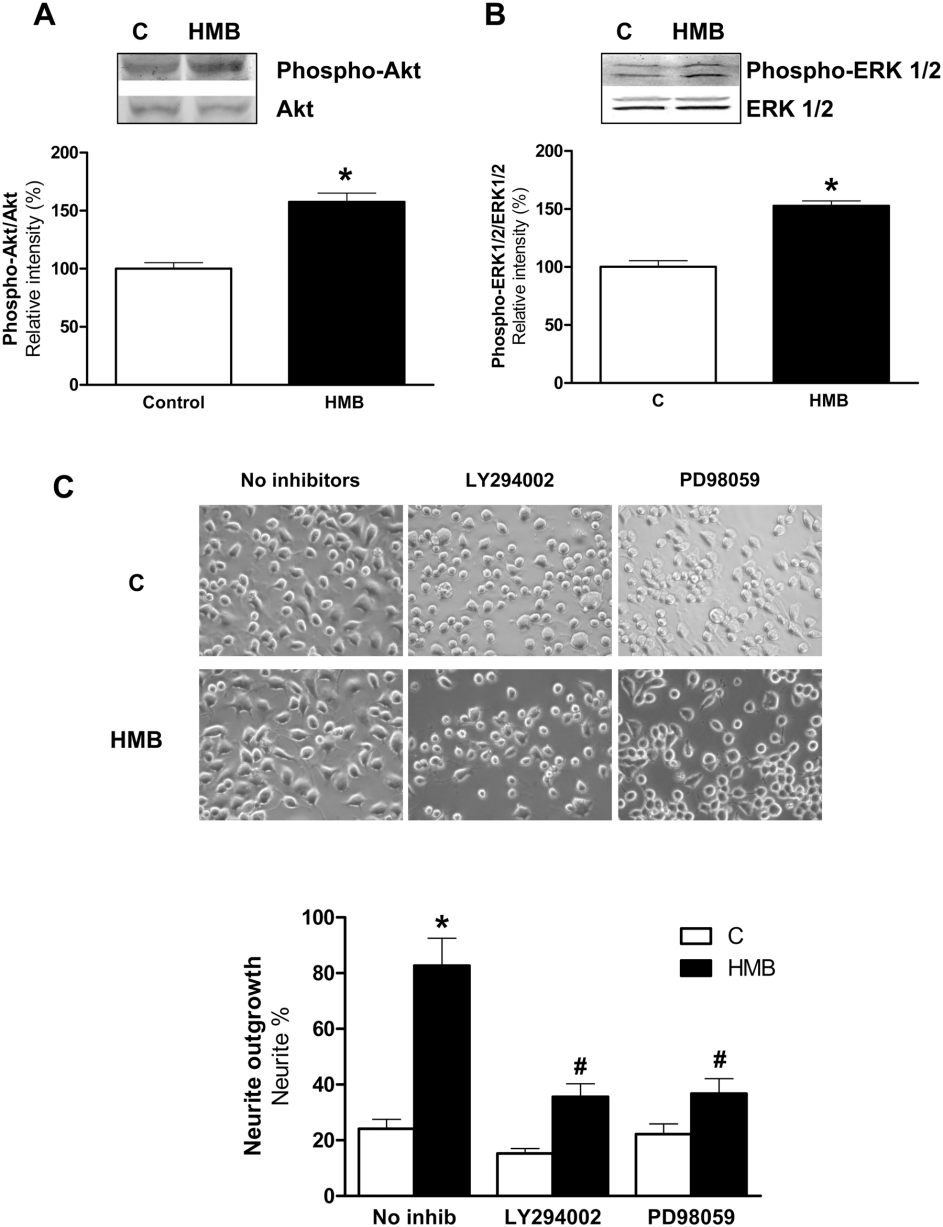
HMB induced neurite outgrowth is mediated by PI3K/Akt and ERK1/2 signaling pathways in Neuro2a cells. Neuro2a cells were treated with 25 μM HMB for 30 min. Western blot analysis was performed using specific antibodies against phospho- and total-antibodies against Akt **(A)** and ERK1/2 **(B)**. **(C)** Neuro2a cells were pre-treated with LY294002 20μM or PD98059 10μM and then treated with 25 μM HMB for 48 h. Inhibitors were maintained during the experiment. Cell morphology was measured as in Fig 1. Results represent means ± SEM (n = 4). * p<0.05 versus control cells. # p<0.05 versus HMB treated cells.

The PI3K/Akt signaling pathway has also been shown to play a key role in the neuronal outgrowth and differentiation induced by substances as neurotrophins, such as nerve growth factor, brain-derived neurotrophic factor and neurotrophin 3 [1], IGF-I [6, 13] or emodin [7]. The involvement of ERK1/2 signaling in neuronal differentiation has been well documented in previous studies. As examples, it has been shown that ERK activation plays an essential role in neurite outgrowth induced by α-lipoic acid [4] or by lithium [5] in Neuro2a cells. HMB stimulates myogenic cell proliferation, differentiation and survival via the PI3K/Akt and MAPK/ERK pathways in muscle cells [18] and improved protein turnover by a PI3K/Akt mechanism [17]. Thus, the expression levels of phosphorylated forms of PKB/Akt (Fig 2A) and ERK1/2 (Fig 2B) were examined by immunoblotting after the treatment of the Neuro2a cells with or without HMB for 30 min. As shown in Fig 2A and 2B, HMB significantly increased the phosphorylation of PKB/Akt and ERK1/2. To elucidate the effect of activation of these kinases by HMB on the regulation of neuritogenesis, Neuro2a cells were pre-incubated with inhibitors of PKB/Akt (20 μM LY294002) and ERK1/2 (10 μM PD98059). The blockade of PKB/Akt or ERK1/2 signaling significantly reduced the effects of HMB in the formation of neurites (Fig 2C). These data provide strong evidence that the activation of both kinases was required for HMB-induced neurite outgrowth.

One of the most important signaling mechanisms downstream of PI3/Akt is mTOR. This protein kinase plays a key role in the control of cell growth, survival and neurite outgrowth [31]. It is activated through phosphorylation in response to growth factors, nutrients, mitogens and hormones [6, 32]. In addition, some studies suggest that pathways linked with mTOR may promote growth and branching in hippocampal neurons [33] memory formation [34] and inhibition of its activity has shown to impair memory consolidation [35]. mTOR activation is regulated mainly by the Akt signaling pathway as well as an alternate pathway regulated by ERK1/2 phosphorylation [32].

**Fig 3:**
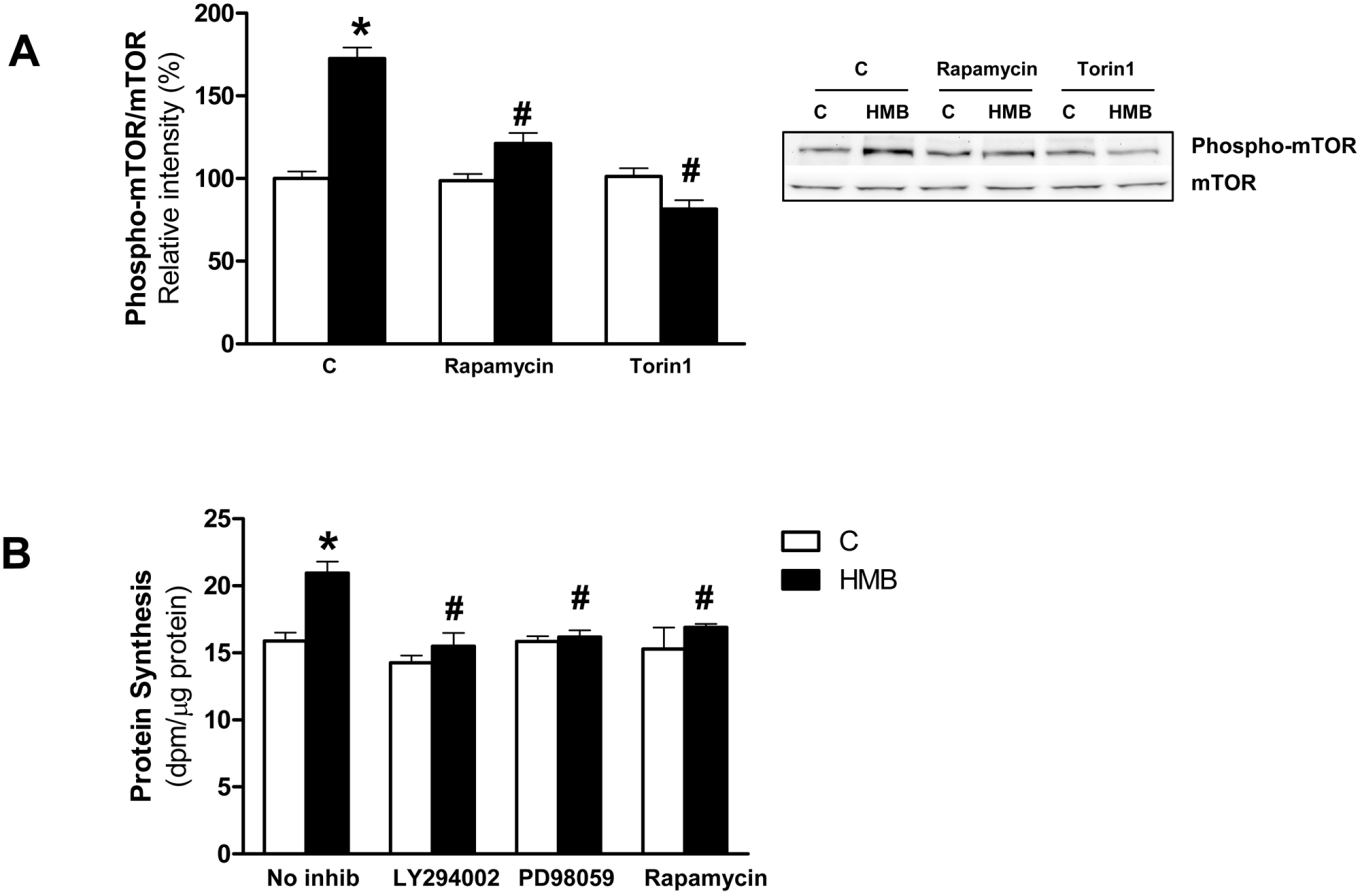
HMB activates mTOR and promotes protein synthesis in Neuro2a cells. **(A)** Neuro2a cells were pre-treated with rapamycin 20nM or Torin1 10 nM and then were treated with 25 μM HMB for 30 min. Western blot analysis was performed using specific antibodies against phospho- and total-antibodies mTOR. **(B)** Protein synthesis was measured in Neuro2a cells incubated with 25 μM HMB for 2 hours in the absence (n = 10) or presence of inhibitors of PI3K/Akt (LY294002 20μM), ERK1/2 (PD98059 10μM) or mTOR (rapamycin 20nM). Pretreatment of inhibitors occurred as in Fig 2. Results represent means ± SEM (n = 4). * p<0.05 versus control cells. # p<0.05 versus HMB treated cells.

To further ascertain the requirement of mTOR pathway activation in HMB-induced neurite outgrowth, mTOR activation in Neuro2a was analyzed by immunoblotting. As expected, HMB significantly increased mTOR phosphorylation in Ser2448, a direct target of PKB/Akt (Fig 3A) and this phosphorylation was blocked by rapamycin and Torin1, two well known inhibitors of the mTOR complex. The role of mTOR in the control of protein synthesis has been widely studied in neuronal cells by its relevance in modulating synaptic plasticity [32]. We tested the dependence of HMB-induced mTOR phosphorylation on protein synthesis. Our results showed that in Neuro2A cells HMB was able to induce a significant increase on protein synthesis (Fig 3B) in similar terms that have been previously described in muscle cells [17, 36]. We next assayed whether the increase on protein synthesis by HMB occurs through a PI3/Akt/mTOR and ERK-coupled mechanisms. Neuro2a were preincubated with the corresponding inhibitors prior treatment with HMB (Fig 3B). Results showed that LY294002, PD98059 and rapamycin blocked the HMB-induced protein synthesis.

**Fig 4:**
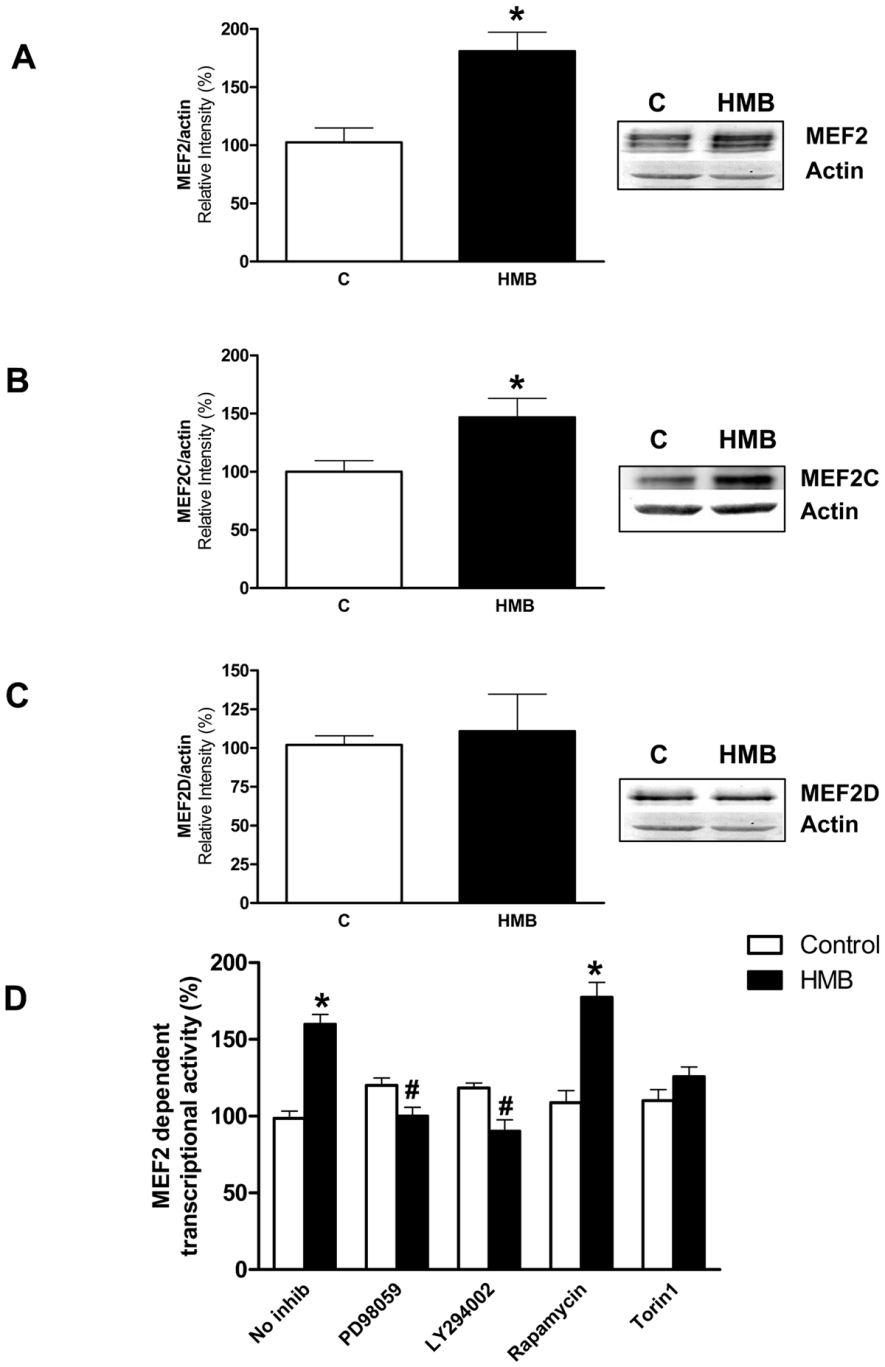
HMB increases MEF2 promoter activity and expression of MEF2 transcription factors. **(A-C)** The expression levels of MEF2 members were analyzed by western-blot using specific antibodies after 24 h incubation with 25 μM HMB (n = 4). **(D)** Cells were transiently transfected with a 4xMEF luciferase reported plasmid to evaluate MEF2-dependent transcription. Cells were pre-incubated for 30 min with 10 μM PD98059, 20 μM LY294002, 20 nM rapamycin or 10 nM Torin1, then incubated with 25 μM HMB for 24 h. Inhibitors were maintained during the experiment (n = 12). Results represent means ± SEM. * p<0.05 versus control cells. # p<0.05 versus HMB treated cells.

In addition, PI3K/Akt and ERK1/2 signaling has been implicated in the regulation of MEF2 proteins [13, 37]. MEF2 constitutes a family of transcription factors including MEF2A, 2B, 2C and 2D [9, 12] that plays a critical role on survival and differentiation of different types of neurons under several experimental conditions [9, 13]. On this sense, we have analyzed the effects of HMB on the expression of MEF2 members by immunoblotting. An antibody called anti-MEF2, recommended for detection of MEF-2A and, to a lesser extent, MEF-2C and MEF-2D was used as well as specific antibodies against MEF-2C and MEF-2D. Our results showed that HMB significantly increased the expression of MEF2A and 2C, but not the expression of MEF2D (Fig 4A–4C).

MEF2C has been reported as crucial for normal neuronal development, distribution and electric activity in the neocortex. In fact, when MEF2C is lacking in early brain development, an abnormal distribution of neurons and developmental anomalies are observed [11]. Moreover, the role of MEF2 isoforms in the brain has been analyzed by studying the synaptic plasticity in the intact neurons in mice in which brain-specific deletion of members of MEF2 family was generated alone or in combination [38]. This study concluded that MEF2C is the major isoform involved in hippocampal synaptic function. In contrast, MEF2A in neurons has not been widely studied. However, several authors suggested that MEF2A may regulate the formation and maintenance of spines in hippocampal and cerebellar neurons and the associated excitatory synapses by modulating the expression of synapse specific genes [38, 39]. Considering the evidence on the role of MEF2A and C on neuron function our results pointed out that HMB could modulate the neural synaptic plasticity.

To determine whether HMB-induced MEF2 activation may be a general mechanism for neurite outgrowth and serve as a convergence point of multiple signaling pathways, we tested the MEF2 dependent transcriptional activity in neural cells treated with PI3/Akt/mTOR and ERK1/2 inhibitors. MEF2 transcriptional activity was increased after HMB incubation (Fig 4D). The blockade of PI3/Akt or ERK1/2 signaling by LY294002 and PD98059 inhibitors respectively confirm the involvement of both signaling pathways on the MEF2 transcriptional activity mediated by HMB. However, inhibition of mTOR through rapamycin did not affect MEF2 transcriptional activity. On the contrary, preincubation with Torin1 inhibited MEF2 transcriptional activity. Rapamycin is an inhibitor of mTORC1 while Torin1 is an inhibitor of mTORC1 and mTORC2. Taking into account that we previously observed an inhibitory effect by rapamycin on protein synthesis in these cells, interestingly, this result could suggest different roles of mTOR promoting neurite outgrowth.

**Fig 5:**
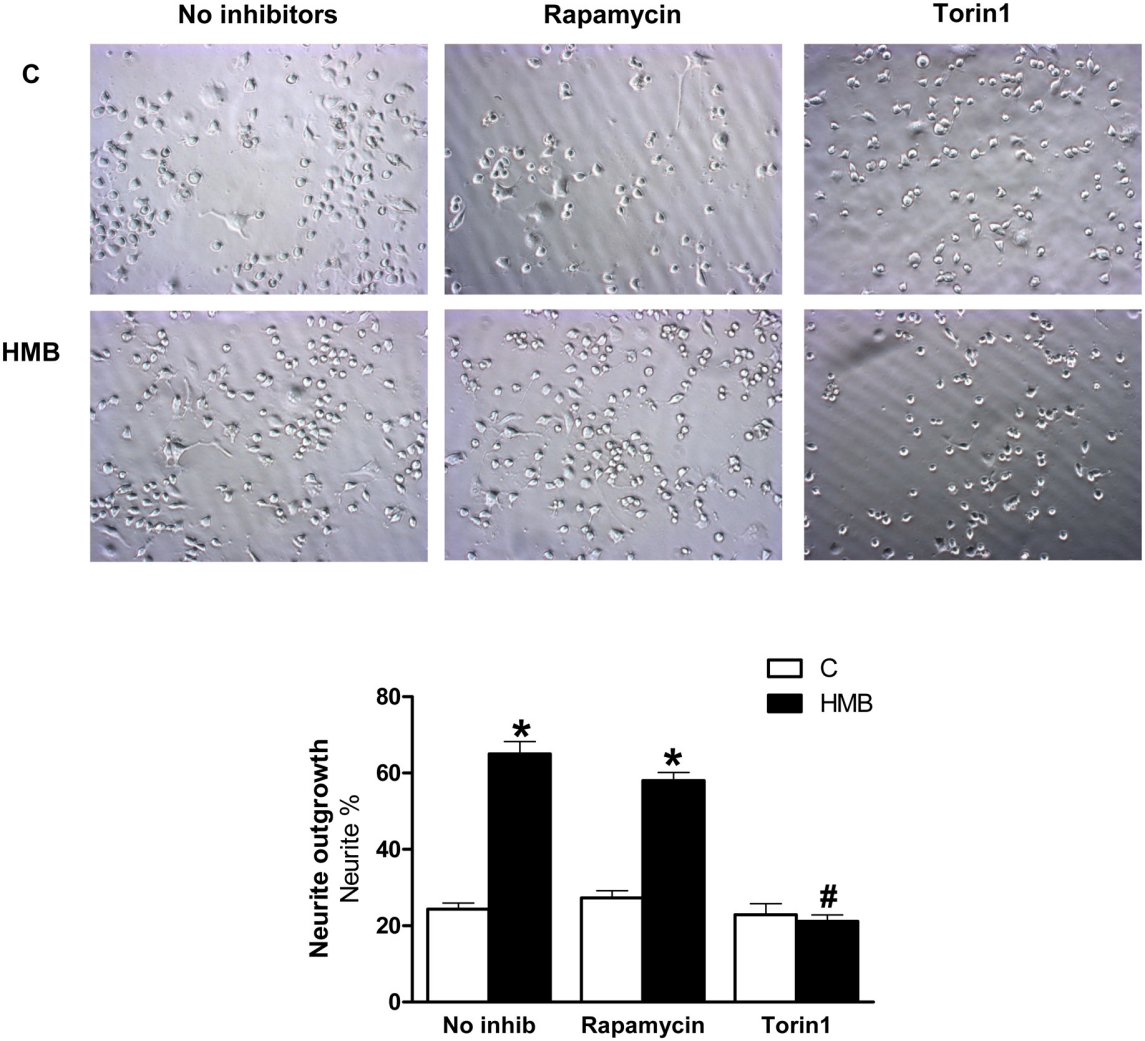
Rapamycin and Torin1 effects on neurite outgrowth in presence of HMB. Cells were pre-treated with rapamycin 20nM or Torin1 10 nM and then treated with 25 μM HMB for 48 h. The inhibitor was maintained during the experiment. Results represent means ± SEM (n = 8). * p<0.05 versus control cells. # p<0.05 versus HMB treated cells.

To clarify the above issue, we preincubated Neuro 2a with rapamycin or Torin1 (Fig 5). The presence of rapamycin did not change the effects of HMB on neurite outgrowth, while incubation with Torin1 blocked neurite formation in the presence of HMB. These findings suggest different functions for mTOR complexes with respect to the regulation of downstream effectors and synaptic plasticity. In fact, it has been reported that while rapamycin-sensitive mTOR complex 1 (mTORC1) has a crucial role in the control of protein synthesis, the rapamycin-insensitive mTOR complex 2 (mTORC2) has an important role in the maintenance of the actin cytoskeleton and has been implicated in the morphological regulation of neurite [32]. In agreement with these findings, HMB may have a complex role modulating neurite outgrowth and synaptic plasticity through both mTORC1 and mTORC2.

In addition, mTOR signaling in neurons seems to be sensitive to energy status. Moreover, recent studies have indicated that insulin-stimulated PI3K/Akt signaling regulates glucose metabolism in the brain, plays important roles in neural development and neuronal activities and affects learning and memory ([40]). GLUT1 and GLUT3 are the main glucose transporters expressed in brain. GLUT1 is detected in the whole brain as two molecular weight forms of 55 kDa and 45 kDa, which are encoded by the same gene but differ in their degree of glycosylation [41]. GLUT3 is mainly localized in neurons [42] and is the most abundant glucose transporter in the brain having five times higher transport capability than GLUT1 [43]. During cerebral development, the increase in GLUT3 expression leads to the expression of the glial 45 kDa GLUT1 and is parallel with neuronal maturation, synaptogenesis, functional activity, and increased rates of cerebral glucose consumption. It has also been described that GLUT3 expression can be increased when neurons are differentiated in vitro [44].

**Fig 6:**
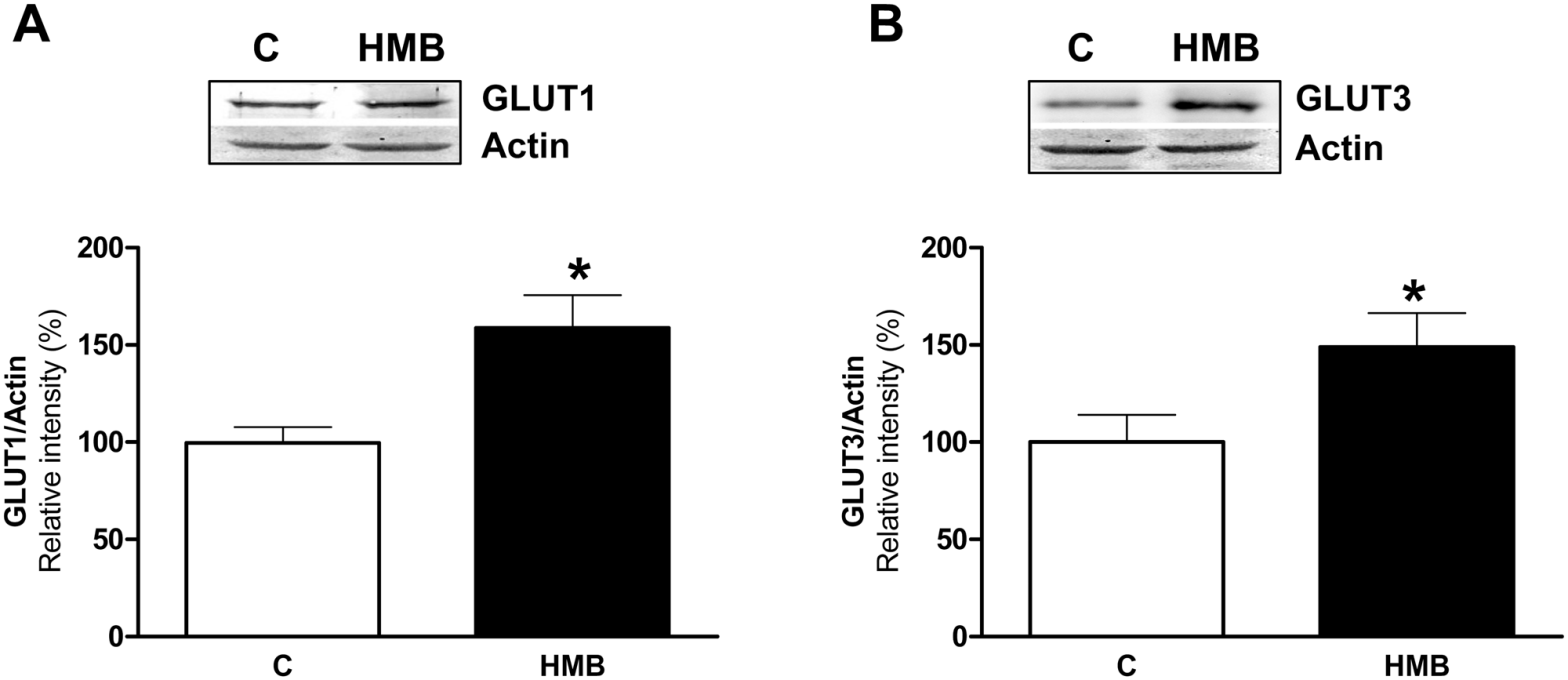
HMB increases the expression of brain glucose transporters. Neuro2a cells were incubated for 24 h with or without 25 μM HMB. Western blot analysis was performed using specific antibodies against GLUT1 and **(A)** and GLUT3 **(B)**. Actin was used as a control for equal loading. Results are expressed as means ± SEM (n = 4). * p<0.05 versus control cells.

Thus, the expression of glucose transporters was analyzed in untreated or HMB-treated Neuro2a cells by immunoblotting. The levels of both GLUT1 (Fig 6A) and GLUT3 (Fig 6B) were increased after treatment with HMB. This result was in concordance with the previous studies in which higher expression of both glucose transporters has been reported when neurons undergo differentiation [42, 44]. Moreover, it supported the role of HMB increasing neurite outgrowth through an increase in the metabolic rate of the HMB treated cells.

## Conclusion

In conclusion, we have shown for the first time that HMB promoted neurite outgrowth through PI3K/Akt and ERK1/2 signaling pathways in Neuro2a cells. Its effect in neuron differentiation is concomitant with higher levels of glucose transporters, the activation of mTOR by mTORC2 and consequently an increase in protein synthesis. Moreover, HMB is involved in promoting MEF2 activity and expression of members of this family of transcriptional factors. We believe that HMB may have great potential as neutrophic factor promoting neuron differentiation and plasticity. Our results indicated a novel effect of HMB on neurite outgrowth and call to further studies to reveal its positive influences on cognitive outcomes.
